# Pharmacokinetic Modeling of an Induction Regimen for *In Vivo* Combined Testing of Novel Drugs against Pediatric Acute Lymphoblastic Leukemia Xenografts

**DOI:** 10.1371/journal.pone.0033894

**Published:** 2012-03-29

**Authors:** Barbara Szymanska, Urszula Wilczynska-Kalak, Min H. Kang, Natalia L. M. Liem, Hernan Carol, Ingrid Boehm, Daniel Groepper, C. Patrick Reynolds, Clinton F. Stewart, Richard B. Lock

**Affiliations:** 1 Children's Cancer Institute Australia for Medical Research, University of New South Wales, Sydney, Australia; 2 Texas Tech University Health Sciences Center, Lubbock, Texas United States of America; 3 Department of Pharmaceutical Sciences, St. Jude Children's Hospital, Memphis, Tennessee, United States of America; Clinica Universidad de Navarra, Spain

## Abstract

Current regimens for induction therapy of pediatric acute lymphoblastic leukemia (ALL), or for re-induction post relapse, use a combination of vincristine (VCR), a glucocorticoid, and l-asparaginase (ASP) with or without an anthracycline. With cure rates now approximately 80%, robust pre-clinical models are necessary to prioritize active new drugs for clinical trials in relapsed/refractory patients, and the ability of these models to predict synergy/antagonism with established therapy is an essential attribute. In this study, we report optimization of an induction-type regimen by combining VCR, dexamethasone (DEX) and ASP (VXL) against ALL xenograft models established from patient biopsies in immune-deficient mice. We demonstrate that the VXL combination was synergistic *in vitro* against leukemia cell lines as well as *in vivo* against ALL xenografts. *In vivo*, VXL treatment caused delays in progression of individual xenografts ranging from 22 to >146 days. The median progression delay of xenografts derived from long-term surviving patients was 2-fold greater than that of xenografts derived from patients who died of their disease. Pharmacokinetic analysis revealed that systemic DEX exposure in mice increased 2-fold when administered in combination with VCR and ASP, consistent with clinical findings, which may contribute to the observed synergy between the 3 drugs. Finally, as proof-of-principle we tested the *in vivo* efficacy of combining VXL with either the Bcl-2/Bcl-xL/Bcl-w inhibitor, ABT-737, or arsenic trioxide to provide evidence of a robust *in vivo* platform to prioritize new drugs for clinical trials in children with relapsed/refractory ALL.

## Introduction

Leukemia is the most common childhood malignancy, accounting for a third of all pediatric cancers and ALL comprises approximately 80% of all leukemia cases in children [1] The prognosis for children diagnosed with ALL has improved markedly during the past 50 years, and current protocols utilizing VCR, a glucocorticoid, and ASP to treat ALL result in over 95% of children entering complete remission with 5-year survival rates of approximately 80% [1].

Despite significant improvements in therapy and supportive care, relapsed ALL is the fifth most prevalent pediatric cancer, and ALL remains the most common cause of death from malignancy in children [2], [3]. For those children who suffer an early relapse in the bone marrow, the prospects for long-term survival are dismal, with the best therapeutic option being hematopoietic stem cell transplantation following induction into second remission. However, in some instances, patients are unable to achieve a second remission [4]. Certain ALL subtypes that are associated with specific chromosomal translocations (e.g. t9;22 and t4;11) remain exceptionally difficult to cure [5], [6]. Moreover, current chemotherapy regimens are associated with morbidity and long-term side effects such as infertility, impaired mental and physical development, and a greater risk of cancer later in life [7], [8].

While increases in pediatric ALL cure rates have principally been invoked through a better use of existing drugs and improvements in supportive care, dozens of new drugs that are being developed primarily to treat adult cancers are potentially available for pediatric clinical trials. However, neither sufficient numbers of pediatric patients are available to test all of these new drugs, nor it is ethical to conduct such trials without strong supporting preclinical data. There is evidence to suggest that future ALL treatment protocols will incorporate new agents into established therapies [9] emphasizing the need for appropriate preclinical multi-agent chemotherapy models. These experimental models should also be able to assess the effects of novel agents when used in combination with standard induction therapy drugs, either to facilitate induction into second remission prior to hematopoietic stem cell transplantation of chemotherapy refractory patients, or as dose-sparing modalities to reduce the side effects of standard therapy.

The attrition rate of potential anti-cancer drugs entering clinical trial is very high, with one study reporting only 5% of agents gaining US FDA approval in 1991–2000 [10]. While the reasons for drug failure in the clinic are likely to be multifactorial, retrospective analysis of pharmacokinetic and pharmacodynamic parameters comparing pre-clinical and clinical data supports the notion that these are crucial in determining efficacy [11]. The distribution and metabolism of certain drugs in different compartments and organs in experimental animals can differ significantly from that of humans, as noted in studies using cyclophosphamide [12], methotrexate [12], topotecan [13] or irofulven [11]. Therefore, to improve predictability of therapeutic efficacy of drugs in humans, pharmacokinetic studies should be conducted during *in vivo* drug testing in order to assess drug disposition in the experimental animal, and adjustments to the drug dose may be necessary to treat the animal at similar systemic exposures to model those used in the clinic.

The non-obese diabetic/severe combined immunodeficient (NOD/SCID) mouse strain is highly receptive to engraftment of human ALL primary biopsy specimens [14], [15], [16]. Moreover, xenografted human cells infiltrate bone marrow, spleen and liver, and blasts in the peripheral blood (PB) retain the morphological characteristics of the original disease [17], [18]. An additional advantage of the orthotopic NOD/SCID mouse model of ALL is that it allows for monitoring disease burden and response to chemotherapeutic drugs in “real-time” by serial sampling of PB [17], [18], [19]. We have previously reported that the *in vivo* responses of a panel of xenografts established from pediatric ALL biopsy specimens to single-agent VCR or DEX significantly correlated with the clinical outcome of the patients from whom the xenografts were derived [17]. Therefore, this experimental model appears highly relevant for the testing of novel treatment strategies.

The aim of this study was to use the xenograft models of pediatric ALL established as systemic disease in NOD/SCID mice in order to: A) use an induction-type regimen of VXL combination therapy in order to induce partial remissions in aggressive and chemoresistant xenografts; B) simultaneously analyze the pharmacokinetics of these drugs (as single agents and in combination) in the NOD/SCID mouse to ensure the clinical relevance of these treatments; and C) assess *in vivo* interactions between VXL and the BH3 mimetic ABT-737 [20] or arsenic trioxide (ATO) with the objective of validating this model for the evaluation of additional compounds in combination with the VXL backbone to facilitate decision making for their incorporation into induction and re-induction protocols and/or dose-sparing regimens.

## Results

### Synergy of VXL combination against ALL cell lines *in vitro*

**Figure 1:**
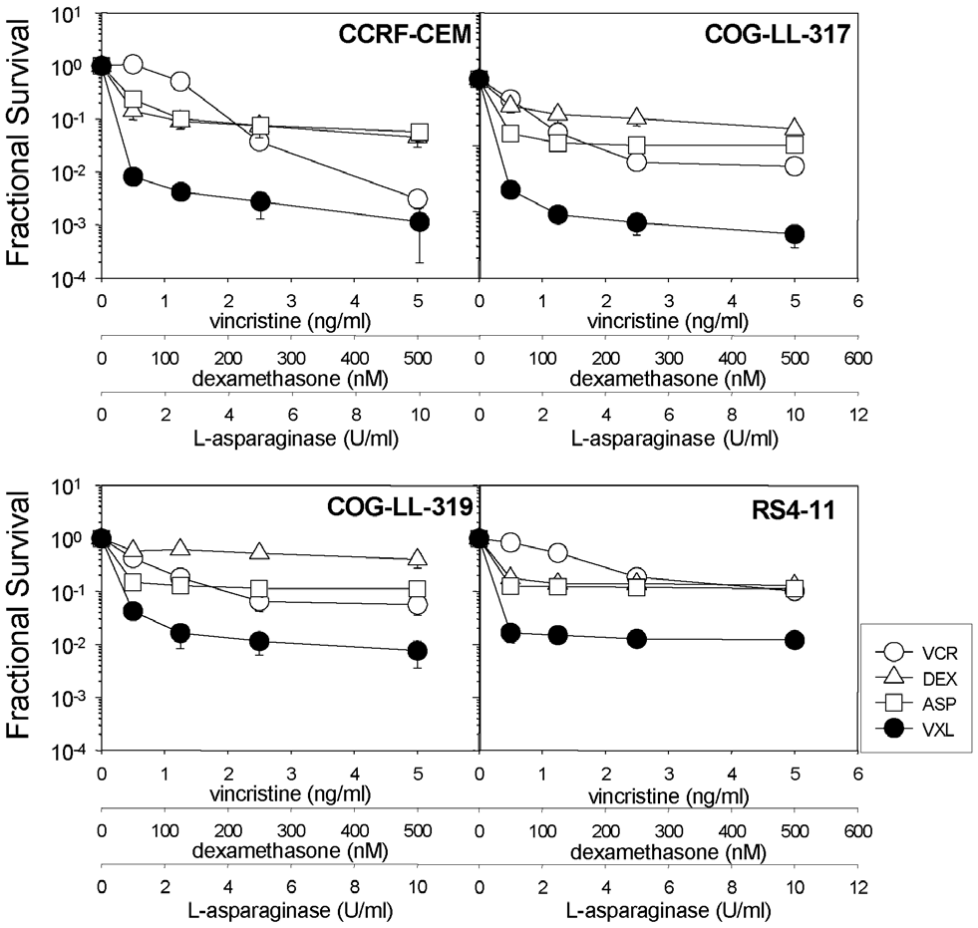
Synergy between VCR, DEX and ASP against ALL cell lines *in vitro*. Cell lines were exposed to VCR (open circles), DEX (open triangles), ASP (open squares), or the triple combination VXL (closed circles), at fixed ratios, and dose-responses were assessed using the DIMSCAN assay as described in Materials and methods. Fractional survival of treated vs. untreated control cells is shown. Each condition included 12 replicates and error bars represent standard deviation. The data shown are representative of two independent experiments.

In order to assess the interactions between VCR, DEX and ASP against ALL cell lines *in vitro*, we examined the cytotoxicity profiles of 4 ALL cell lines (CCRF-CEM, COG-LL-317, COG-LL-319 and RS4-11) exposed to VCR, DEX, ASP and the triple-drug combination (VXL) (Figure 1). Although the ALL cell lines exhibited varied sensitivity to VCR, DEX, and ASP, the combination of the 3 drugs consistently displayed synergistic interactions at all drug concentrations tested, with the only exception being the CEM cell line exposed to the highest drug concentrations (Figure 1 and Table 1). With this minor exception aside, the calculated CI values indicate strong synergy between the 3 drugs *in vitro* with a common trend towards stronger synergy at lower combination concentrations for the cell lines CEM, COG-LL-317 and COG-LL-319 (Table 1).

**Table 1:** Combination Indices of *in vitro* cytotoxicity assays.

VCR [ng/mL]	DEX [nM]	ASP [U/mL]	Combination Index Values			
			CEM	COG-LL-317	COG-LL-319	RS4-11
0.5	50	1	0.15	0.10	0.10	0.04
1.25	125	2.5	0.33	0.11	0.11	0.09
2.5	250	5	0.61	0.15	0.15	0.16
5	500	10	1.04	0.21	0.21	0.10

VCR, vincristine; DEX, dexamethasone; ASP, l-asparaginase.

### Real-time monitoring of leukemia engraftment and response to therapy

Figure S1 represents leukemia infiltration of bone marrow, spleen, liver and PB at weekly intervals following inoculation of NOD/SCID mice with xenograft ALL-19, and confirms that monitoring %huCD45^+^ cells in the PB provides a reliable representation of overall leukemic burden in the animal, in agreement with a previous report [19]. Engraftment and response to therapy in all subsequent experiments were monitored by weekly enumeration of the %huCD45^+^ cells in the PB.

### Optimization of the VXL regimen using *in vivo* childhood ALL xenograft models

We have previously determined the *in vivo* VCR and DEX sensitivities of a panel of childhood ALL continuous xenografts derived from primary patient biopsies (details of patient characteristics are included in Table S1) [17]. In order to develop combination chemotherapy protocols that mimic induction regimens administered to pediatric ALL patients, it was also necessary to determine the *in vivo* efficacy of ASP as single agent. For these and subsequent experiments ASP was administered Mon-Fri for 4 weeks in an attempt to mimic the dosing schedule administered to patients. ASP at a dose of 2500 U/kg delayed the progression of ALL-3 by approximately 28 days (Figures S2A and D, Table S2) but had no effect against ALL-7 or ALL-19 (Figures S2B–D, Table S2). ALL-7 and ALL-19 were previously shown to be relatively resistant to DEX and VCR *in vivo* [17], and are derived from patients who succumbed to their disease at 13 and 11 months following diagnosis, respectively (Table S1).

**Figure 2:**
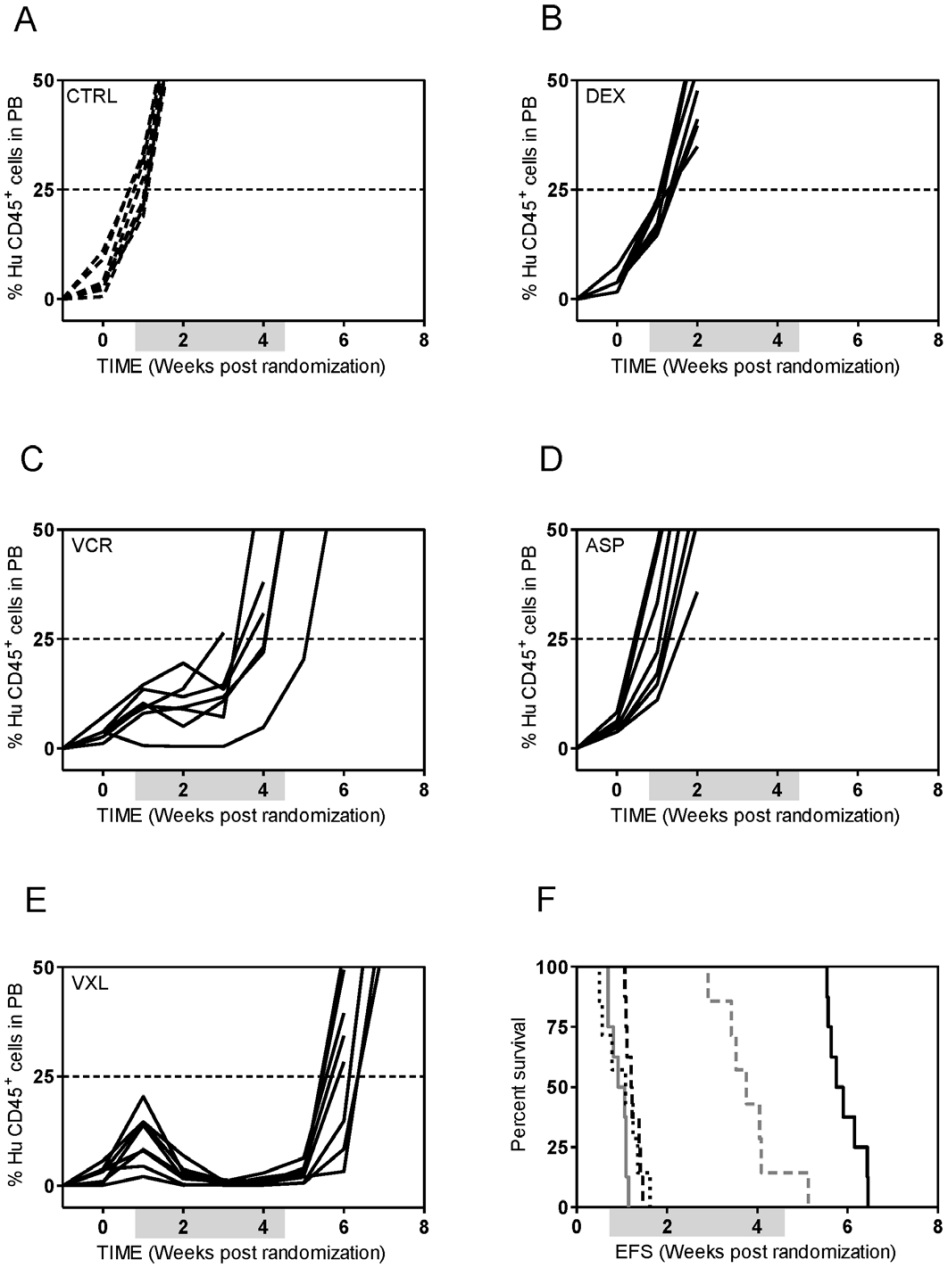
*In vivo* sensitivity of ALL-19 to low dose VCR, DEX and ASP. Female mice were engrafted with ALL-19 cells and treated with vehicle (**A**); DEX (5 mg/kg) (**B**); VCR (0.15 mg/kg) (**C**); and ASP (1000 U/kg) (**D**); as single agents or the combination of the three drugs at the same doses (VXL) (**E**). The %huCD45^+^ cells in PB of individual mice (**A–E**); control vehicle-treated mice (dashed lines); drug-treated mice (solid lines). Kaplan-Meier analysis of EFS (**F**) control (grey solid line), VCR (grey dashed line), DEX (black dashed line), ASP (black dotted line), VXL (solid black line). All events were leukaemia-related. Shaded boxes represent the treatment period.

We next examined the efficacy of VCR (0.25 mg/kg), DEX (7.5 mg/kg) and ASP (2500 U/kg) as single agents and in combination against ALL-7 and ALL-19. Despite being attenuated to a quarter of the maximum tolerated dose (MTD) VCR still effectively delayed leukemia progression compared to control mice by approximately 5 weeks in both xenografts (Figures S3A and E, Table S2). At this dose DEX showed modest efficacy against ALL-7, delaying its progression by approximately 4 weeks while ASP was ineffective. However, the VXL combination treatment of ALL-7 resulted in a LGD of 82.8 days, 18.5 days greater than the sum of the LGDs for the individual drugs (Table S2). Similarly, while single agents DEX and ASP remained ineffective in delaying the progression of ALL-19, the VXL combination treatment resulted in a LGD of 47.5 days, which was 11 days greater than the sum of the LGDs for the individual drugs (Figures S3B, C and E, Table S2). Unexpectedly, some of the mice in the VXL treated group experienced toxicity, with only 3 mice reaching leukemia-related events, while some mice treated with ASP alone also exhibited mild weight loss. Therefore, and in consideration of the aims of the study, the VCR, DEX and ASP doses were further attenuated to 0.15 mg/kg, 5 mg/kg and 1000 U/kg, respectively. VCR at a dose of 0.15 mg/kg was still effective against ALL-19 with a LGD of 19.4 days (Figure 2C, Table S2); DEX and ASP were ineffective as single agents at the attenuated doses (Figures 2B and D, Table S2). Importantly, these doses of DEX and ASP effectively delayed the progression of the chemosensitive xenograft ALL-3 (data not shown and Table S2). The VXL combination, which was well tolerated, delayed the progression of ALL-19 by 33.9 days, which was 12.2 days greater than the sum of the LGDs for the individual drugs (Figures 2E and F, Table 2 and Table S2). In contrast to previous experiments in which higher drug doses were used and mice were culled due to drug toxicity, all events in this experiment were leukemia related.

**Table 2:** *In vivo* responses of ALL xenografts to ABT-737, VXL or VXL/ABT-737 combination treatments.

Xenograft	Treatment	Median EFS [days] (number of mice)	LGD [days]	Significance vs control [*P* value]	Significance vs VXL [*P* value]
**ALL-2**	Control	19.7 (6)	**-**		
	ABT-737	27.3 (8)	**7.6**	**0.0236**	
	VXL	78.6 (8)	**58.9**	**<0.0001**	
	VXL/ABT-737	99.7 (7)	**80.0**	**0.0002**	**0.0001**
**ALL-3**	Control	15.8 (8)	**-**		
	VXL	>133.4 (8)	**>117.6**	**<0.0001**	
**ALL-4**	Control	10.1 (8)	**-**		
	ABT-737	8.5 (8)	**0**	**0.025**	
	VXL	32.9 (8)	**22.8**	**0.0048**	
	VXL/ABT-737	39.3 (8)	**29.2**	**<0.0001**	**0.0069**
**ALL-7**	Control	12.7 (8)	**-**		
	VXL	58.9 (8)	**46.2**	**<0.0001**	
**ALL-8**	Control	10.9 (8)	**-**		
	ABT-737	10.7 (8)	0	1.0	
	VXL	64.6 (8)	**53.7**	**<0.0001**	
	VXL/ABT-737	76.5 (7)	**76.5**	**0.0001**	**0.0245**
**ALL-10**	Control	12.5 (7)	**-**		
	ABT-737	26.0 (8)	**13.5**	**<0.0001**	
	VXL	71.8 (8)	**59.3**	**<0.0001**	
	VXL/ABT-737	78.3 (8)	**65.8**	**<0.0001**	0.0966
**ALL-11**	Control	19.3 (8)	**-**		
	VXL	119 (10)	**99.7**	**<0.0001**	
**ALL-16**	Control	13.2 (8)	**-**		
	VXL	>160.0 (5)	**>146.8**	**<0.0001**	
**ALL-17**	Control	15.1 (6)	**-**		
	ABT-737	28.9 (8)	**13.8**	**0.0005**	
	VXL	72 (7)	**56.9**	**0.0002**	
	VXL/ABT-737	72 (7)	**56.9**	**0.0002**	0.3389
**ALL-19**	Control	6.9 (8)	**-**		
	VXL	40.8 (7)	**33.9**	**0.0002**	

Significant values are shown in bold.

**Figure 3:**
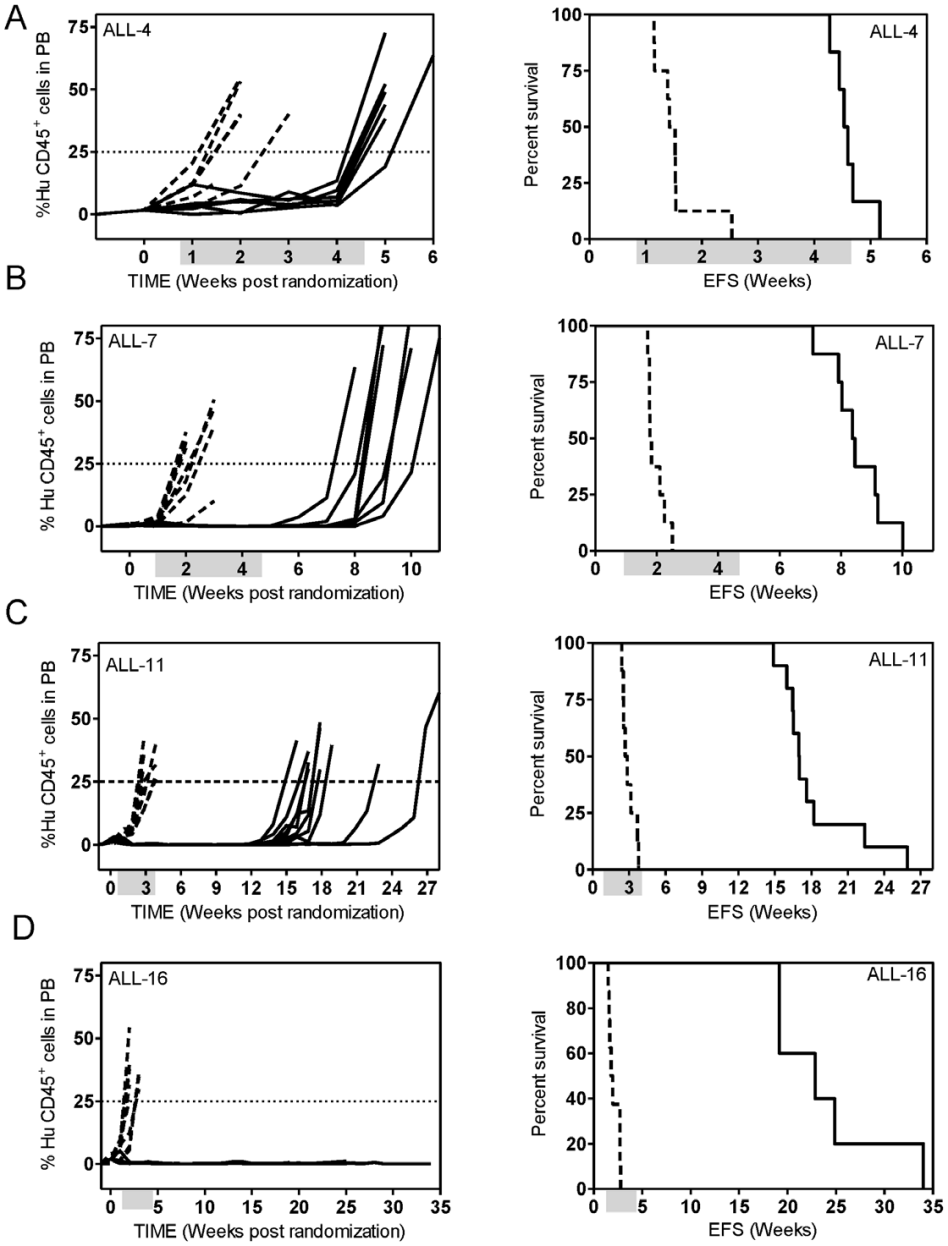
*In vivo* sensitivity of ALL xenografts to VXL combination treatment. Female mice were engrafted with: ALL-4 (**A**); ALL-7 (**B**); ALL-11 (**C**); or ALL-16 (**D**); and treated with a combination of VCR (0.15 mg/kg), DEX (5 mg/kg) and ASP (1000 U/kg). The %huCD45^+^ cells in PB of individual mice (left panel) and Kaplan-Meier analysis of EFS (right panel). Control vehicle-treated mice (dashed lines); drug-treated mice (solid lines). Shaded boxes represent the treatment period. No leukaemia related events were recorded for the drug treated group of ALL-16 engrafted mice.

**Figure 4:**
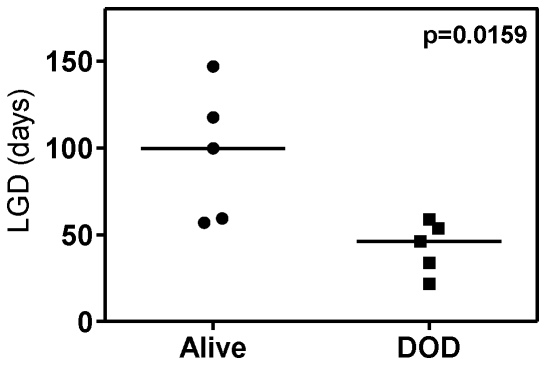
LGD in response to VXL treatment in xenografts stratifies according to patient outcome. Median LGD obtained by VXL treatment for a panel of ALL xenografts derived from long term survivors (Alive) and from patients who died of their disease (DOD). There is evidence that the two groups are different (*p* = 0.0159) by Mann-Whitney two tailed test.

We next examined the effect of the optimized VXL combination treatment against several other previously established BCP-ALL and T-ALL xenografts. As shown in Figures 3 and 4, the responses of the xenografts to the VXL combination treatment varied. The chemosensitive T-ALL xenograft ALL-16 was the most sensitive to treatment with VXL, with a LGD greater than 146.8 days and no leukemia related deaths (Figures 3D, Table 2), while the Philadelphia chromosome-positive ALL-4, was only delayed by approximately 23 days (Figure 3A, Table 2). Other xenografts exhibited delays in leukemia progression intermediate of ALL-16 and ALL-4 (Table 2). Interestingly, we observed that LGDs measured following VXL combination treatment were significantly higher for xenografts derived from long term survivors (median of 99.7 days) than those derived from patients who died of their disease (median of 46.2 days) (*p* = 0.0159, Mann-Whitney test) (Figure 4). Also, by setting an arbitrary cut-off value of 55 days, we obtained evidence of interdependence between the LGD for the xenografts and patient clinical outcome (*p* = 0.047, two sided chi-square contingency test, data not shown).

### Pharmacokinetic analysis of VCR, DEX and ASP in NOD/SCID mice

**Figure 5:**
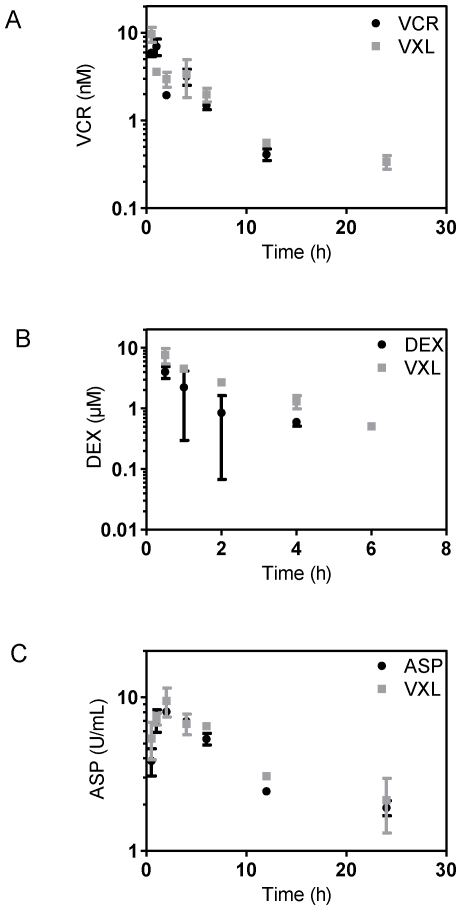
Pharmacokinetic analysis of VCR, DEX and ASP in leukemias bearing NOD/SCID mice. Engrafted female mice (ALL-19) were treated with VCR (0.15 mg/kg), DEX (5 mg/kg), ASP (1000 U/kg) or their combination (VXL) at the same doses. Three mice each were sacrificed at specified time points and drug concentrations in plasma for VCR (**A**); DEX (**B**); and ASP (**C**) in the single agent or combination treatment were assessed as described in Materials and Methods.

We next undertook pharmacokinetic studies to establish whether plasma drug concentrations achieved in mice were clinically relevant and to determine if the disposition of a drug was altered when administered in combination. Mice with established leukemia (ALL-19) were treated with each drug as either a single agent or in the triple combination and pharmacokinetic studies were performed. The concentration-time plot for each drug as a single agent and in combination is presented in Figure 5. For VCR and ASP, a two-compartment model produced a reasonable fit to the data from both single agent and combination groups simultaneously (Figures 5A and 5C). No apparent difference between the single agent and combination groups was noted (Table 3). For DEX, the one compartment pharmacokinetic model adequately described the data shown in Figure 5B. Administration of VXL increased the DEX area under the concentration-time curve (AUC) approximately 2-fold and the C_max_ by 1.5-fold (Table 3).

**Table 3:** Estimated pharmacokinetic parameters.

‡Parameter	Single Agent	VXL Combination
	**VCR 0.15 mg/kg**	**VCR 0.15 mg/kg**
AUC_0-∞_ (mg/L*min)	1.60	1.75
C_max_ (ng/mL)	6.2	8.7
T_max_ (h)	1	0.5
	**DEX 5 mg/kg**	**DEX 5 mg/kg**
AUC_0-∞_ (ng*h/mL)	3279.05	6792.95
C_max_ (ng/mL)	1760.73	2849.02
T_max_ (h)	0.16	0.25
	**ASP 1000 U/kg**	**ASP 1000 U/kg**
AUC_0-∞_ (U*h/mL)	87.02	99.44
C_max_ (U/mL)	8.27	8.90
T_max_ (h)	2.21	1.97

‡ Abbreviations. AUC_0-∞_: Area under the concentration-time curve from zero to infinity; C_max_: actual maximum concentration observed after drug administration; T_max_: time of maximum drug concentration.

### VXL treatment regimen as a platform for the detection of synergy in combination with novel drugs against pediatric ALL

**Figure 6:**
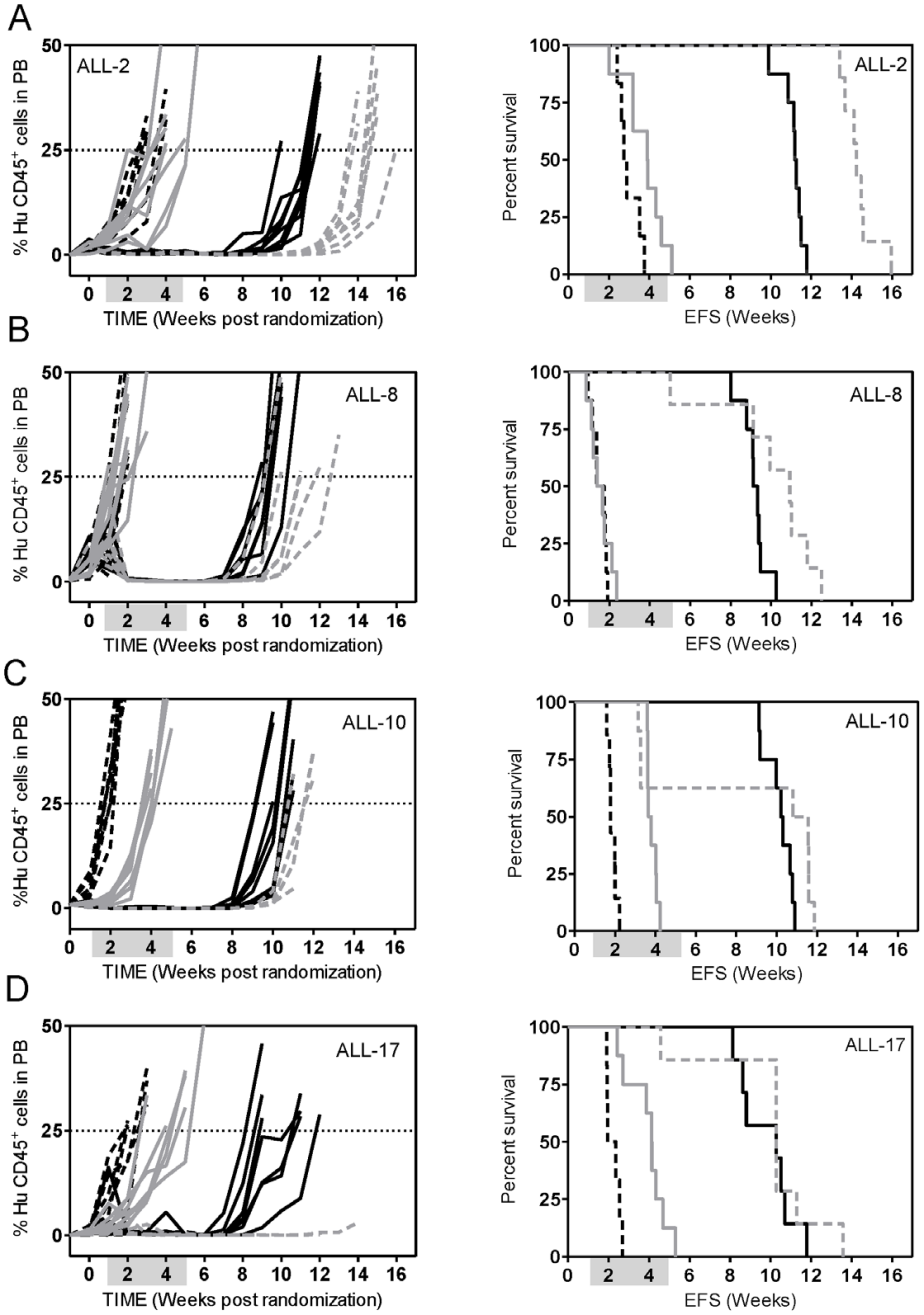
*In vivo* sensitivity of ALL xenografts to VXL and VXL/ABT-737 combination treatments. Female mice were engrafted with: ALL-2 (**A**); ALL-8 (**B**); ALL-10 (**C**); or ALL-17 (**D**) and treated with a diluent vehicle (controls, dashed black lines), ABT-737 (25 mg/kg, solid grey lines), VXL combination (solid black lines), or VXL+ABT-737 quadruple combination (dashed grey lines). Engraftment kinetics indicated by %huCD45^+^ cells in PB of individual mice (left panel) and Kaplan-Meier survival curves (EFS) (right panel) are shown. Shaded boxes represent the treatment period. All events were leukemia-related except for 1 and 4 in the VXL/ABT-737-treated group of the ALL-8, and ALL-10, respectively. In the ALL-17 quadruple drug combination cohort all mice were culled due to leukemia or toxicity unrelated morbidity.

The VXL treatment was optimized so that additional drugs could be used in combination with this platform in order to model interactions with the induction-type regimen typical of ALL therapy in the clinical setting. For this purpose we first selected ABT-737, a BH3 mimetic, shown to inhibit the pro-survival function of Bcl-2, Bcl-xL and Bcl-w and to induce apoptosis in a variety of cancer cell types including leukemias [20], [21], [22]. We have previously shown that ABT-737 potentiated the effect of the VXL treatment [23] and interacted synergistically with ASP/topotecan combination against chemoresistant xenografts ALL-7 and ALL-19 [24]. In the present study we tested the VXL/ABT-737 combination against 3 additional chemoresistant ALL xenografts (ALL-2, ALL-4 and ALL-8; derived from patients who died of their disease) and 2 xenografts of intermediate chemosensitivity (ALL-10 and ALL-17; derived from patients who are currently in remission, Table S1). This drug combination could not be tested on the other 3 highly chemosensitive xenografts derived from long-term survivors (ALL-3, ALL-11 and ALL-16) because of the high efficacy of the VXL treatment alone. ABT-737 significantly potentiated the effect of the VXL combination treatment in the chemoresistant ALL xenografts, from 6.4 days against ALL-4 to 13.5 days against ALL-2, above what was predicted if the effects were additive (Figure 6, Table 2). No correlations (Spearman) have been found between the Bcl-2 proteins expression and response to the VXL/ABT-737 combination treatment.

**Figure 7:**
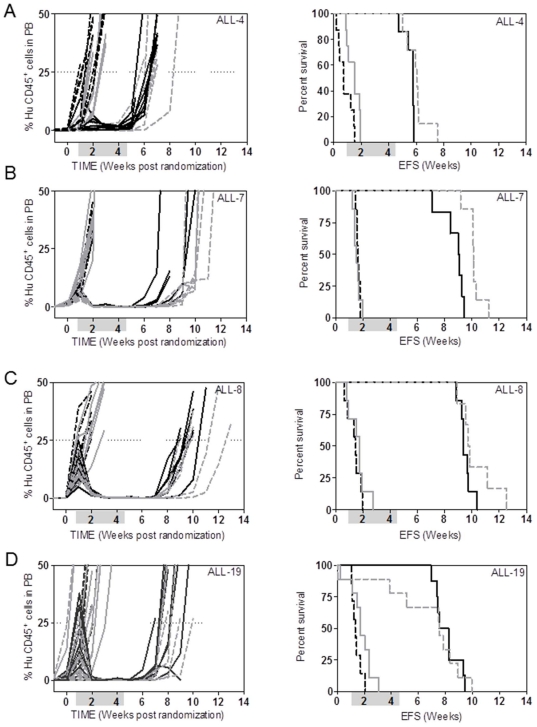
*In vivo* sensitivity of ALL xenografts to VXL and VXL/ATO combination treatments. Female mice were engrafted with: ALL-4 (**A**); ALL-7 (**B**); ALL-8 (**C**); or ALL-19 (**D**) and treated with a diluent vehicle (controls, dashed black lines), ATO (2.5 mg/kg, solid grey lines), VXL combination (solid black lines), or VXL+ATO quadruple combination (dashed grey lines). Engraftment kinetics indicated by %huCD45^+^ cells in PB of individual mice (left panel) and Kaplan-Meier survival curves (EFS) (right panel) are shown. Shaded boxes represent the treatment period.

The VXL therapy was also combined with ATO, a standard chemotherapeutic agent used in the treatment of acute promyelocytic leukemia, since a previous study suggested that ATO reverses DEX resistance in ALL [25]. As demonstrated in Figure 7 and Table 4 ATO as a single agent had a small but statistically significant effect in delaying the progression of only one (ALL-4) of four ALL xenografts tested. When combined with VXL, ATO significantly improved the progression delays of two (ALL-4 and ALL-7) of four ALL xenografts tested, although the augmentation of VXL efficacy by ATO appears unlikely to be of biological significance.

**Table 4:** *In vivo* responses of ALL xenografts to ATO, VXL or VXL/ATO combination treatments.

Xenograft	Treatment	Median EFS [days] (number of mice)	LGD [days]	Significance vs control [*P* value]	Significance vs VXL [*P* value]
**ALL-4**	Control	5.1 (8)	**-**		
	ATO	10.5 (8)	**5.4**	**0.0075**	
	VXL	40.3 (7)	**35.2**	**0.0001**	
	VXL/ATO	42.3 (7)	**37.2**	**0.0001**	**0.0252**
**ALL-7**	Control	11.5 (7)	**-**		
	ATO	10.8 (7)	0	0.6734	
	VXL	63.3 (6)	**51.8**	**0.0004**	
	VXL/ATO	70.8 (7)	**59.3**	**0.0001**	**0.0012**
**ALL-8**	Control	10.3 (7)	**-**		
	ATO	12.4 (7)	2.1	0.6399	
	VXL	65.6 (7)	**55.3**	**0.0001**	
	VXL/ATO	68.4 (6)	**58.1**	**0.0004**	0.1499
**ALL-19**	Control	9.3 (7)	**-**		
	ATO	11.9 (9)	2.6	0.115	
	VXL	55.2 (8)	**45.9**	**0.0270**	
	VXL/ATO	52.9 (9)	**43.6**	**0.0015**	0.9394

Significant values are shown in bold.

## Discussion

This study reports the optimization, based on pharmacokinetic parameters, of an induction-type regimen for the preclinical prioritization of new anti-cancer agents in combination with established drugs in pediatric ALL. This platform will also be useful to eliminate new drugs that are unlikely to provide any benefit in the clinical management of ALL, and thereby avoid the unnecessary treatment of pediatric patients. Desirable characteristics of a preclinical drug testing xenograft model include that it: (1) represents the human disease phenotypically and genetically; (2) reflects the heterogeneity of the clinical disease; (3) exhibits high take rates of engraftment and reproducible leukemia progression within treatment groups; (4) is amenable to routine monitoring of leukemia progression during and after drug treatment; (5) reflects clinically relevant responses to established chemotherapeutic drugs; and (6) takes into consideration differences in pharmacokinetics of drugs between the selected species and humans and thus assesses clinically equivalent doses, since increased host tolerance leads to overestimation of drug efficacy and *vice-versa*. The panel of pediatric ALL xenografts previously described by our group adheres favorably to these criteria [17], [18], [26]. In the current study we extend the utility of this model to multi-agent chemotherapy consisting of a VXL induction-type regimen, and demonstrate its usefulness for testing novel drugs, which could be incorporated into induction/re-induction phases of treatment in order to improve therapeutic outcome for high risk ALL patients. A previous study by Ek *et al.* which used VCR/methylprednisolone/ASP in combination to assess the efficacy of the anti-CD19 immunotoxin B43-PAP in a preclinical model of ALL demonstrated the usefulness of testing new agents in combination with established drugs. However, the clinical relevance of the doses used in that model was unclear [27].

In this study we demonstrated that the cytotoxic effect of VXL combination treatment was highly synergistic against ALL cell lines *in vitro* and that this synergy was maintained over a broad range of drug concentrations. More importantly, using these ALL xenograft models we have shown that *in vivo* doses of DEX and ASP that, individually, caused no delay in progression of aggressive and chemoresistant xenografts (ALL-7 and ALL-19), resulted in synergistic interactions when combined with VCR. The reason for the observed synergy is presently unknown, however it could be explained, at least in part, by the finding that the combination of DEX with VCR and ASP resulted in a significantly higher exposure to DEX when compared to DEX alone, when administered at the same dose. This observation is consistent with a recently published study that examined DEX pharmacokinetics in pediatric ALL patients [28]. The authors have reported that while DEX pharmacokinetics were highly varied between patients, co-administration of ASP decreased clearance of DEX resulting in the increased systemic exposure to the drug [29]. The mechanism responsible for decreased clearance of DEX is not clear but is positively correlated with levels of serum albumin.

In addition, the synergistic effect of the VXL combination may also occur at the cellular level, where it could be due to the non-overlapping mechanisms of cytotoxicity and/or resistance to the 3 drugs. VCR binds to the β subunit of the α/β-tubulin heterodimer and suppresses microtubule dynamics, blocks cell cycle progression at the G_2_/M phase and induces apoptosis [30]. A common mechanism of VCR resistance involves reduced intracellular drug accumulation due to overexpression of multidrug transporters such as MDR1 and MRP1 [31]. Glucocorticoid-induced apoptosis of lymphocytes is mediated via the cytoplasmic glucocorticoid receptor (GR), and resistance is frequently associated with mutation or decreased expression of the GR, or of interruption of downstream apoptotic pathways [32], [33]. ASP catalyzes the hydrolysis of l-asparagine to l-aspartic acid and ammonia, which results in depletion of serum asparagine and starvation of ALL cells by depriving them of an essential amino acid [34]. Increased expression of asparagine synthetase protein appears to be one mechanism of ALL resistance to ASP [35].

We have previously shown that the response of a panel of xenografts to single-agent DEX (15 mg/kg) and VCR (0.5 mg/kg), at higher doses than those used in this study, correlated well with patient outcome [17]. Moreover, ALL-7 and ALL-19, which were derived from patients who died from their disease within 13 months of diagnosis, were inherently more resistant to both drugs when compared to ALL-3, which was derived from a patient who remains in remission more than 17 years following diagnosis. An interesting finding of the current study is that the magnitude of the response to the VXL combination therapy also correlates with patient outcome. The median LGD recorded for a cohort of xenografts derived from long term survivors was over 2-fold higher than that observed for xenografts derived from patients who died of their disease. This result provides further evidence to support the clinical relevance of this pre-clinical ALL xenograft model. However, it should be noted that the cohort of xenografts used in this study was small and heterogeneous, and that the patients from whom the xenografts were derived were not always treated on identical induction protocols. Therefore, further validation using a larger cohort of xenografts derived from patients treated on the same protocol is warranted.

While VCR, DEX and ASP have been in clinical use for almost 50 years, comprehensive pharmacokinetic studies of these drugs in patients are lacking. Furthermore, pharmacokinetic data of these drugs in murine models are even scarcer. Retrospective analysis of systemic exposures from preclinical and clinical data suggests that these are crucial in determining drug efficacy. Due to a lack of pharmacokinetic data it is a common practice to evaluate efficacy of drugs at their MTD rather than at clinically equivalent doses. However, differences in absorption, distribution, metabolism and excretion of drugs could vary considerably between species, resulting in different systemic exposures to a drug, thus affecting therapeutic efficacy. Frequently, the MTD of drugs are higher for mice than for humans [12], [13] and consequently pre-clinical models are often criticized for overestimating the efficacy of chemotherapeutic agents. Therefore, in order to confirm the clinical relevance of this induction type regimen it was important to establish whether the plasma drug exposures in mice were comparable to those achievable in humans. Studies in pediatric ALL patients have revealed that the systemic exposure to VCR following treatment at 2.0 mg/m^2^ as an intravenous bolus varied considerably among patients ranging from 0.9 to 14.9 mg/L*min in one study [36] and from 0.4–7.5 mg/L*min in another study [37] with median exposures of 5.4 and 2.8 mg/L*min, respectively. Even though in our study a single dose of VCR (0.15 mg/kg) administered to female NOD/SCID mice engrafted with a BCP-ALL xenograft resulted in a lower median systemic exposure to this drug (1.6 mg/L*min) than those reported in humans, this value falls well within the range recorded for patients with ALL.

Reported pharmacokinetic parameters for DEX vary considerably between studies. In pediatric patients a moderate dose of 8 mg/m^2^/day resulted in mean AUC value of 167 ng/mL*h [28]. In other studies in adults the AUC values were not reported, however from their pharmacokinetic parameters we can estimate that a single dose of 20 mg DEX intravenously (i.v.) or 300 mg DEX administered orally resulted in systemic exposures of 2000 ng/mL*h [38] and 8000 ng/mL*h, respectively [39]. Overall we conclude that systemic exposure to DEX in our model falls within the upper range of what has been reported in patients.

The dose, schedule, preparation and the route of administration of ASP, all of which influence pharmacokinetics and pharmacodynamics of the drug, vary considerably between protocols used in the clinic to treat pediatric ALL patients. Furthermore, since ASP is immunogenic, the presence or absence of anti-ASP antibodies as well as their titer, are known to increase the variability of ASP pharmacokinetic profiles in patients. The most commonly used treatment protocols utilize ASP derived from either *Escherichia coli* or *Erwinia chrysanthemi*, which are typically administered i.v. or intramuscularly (i.m.) 2–3 times a week, at doses ranging from 5000–12000 U/m^2^ [40], although protocols using *E. chrysanthemi* derived ASP at doses as high as 30000 U/m^2^ administered daily for 10 days have been reported [41]. A recent clinical trial has demonstrated that following a low dose ASP (5000 U/m^2^) treatment the systemic exposure to the drug varied considerably among patients ranging between 38.6 and 83.8 U*h/mL [42]. The AUC value (87.0 U*h/mL) in our model falls only slightly above this range and since the clinical study used a low dose of ASP we conclude that the systemic exposure to ASP achieved in our preclinical model reflects clinical scenarios.

ABT-737 and the closely related clinical homolog ABT-263 (Navitoclax) are BH3-mimetics, which inhibit the anti-apoptotic Bcl-2 family members, Bcl-2, Bcl-xL and Bcl-w [20], [43]. The activity of both drugs has been extensively evaluated in a variety of preclinical cancer models, including ALL xenografts [20], [21], [23], [24], [43], [44], [45], [46]. These studies have demonstrated that both drugs have potent single agent *in vitro* and *in vivo* activity against a variety of cancer cell lines and primary cells including ALL. Furthermore, both compounds significantly potentiate the efficacy of established and novel chemotherapeutic agents. ABT-263 is currently being evaluated in phase 1/2 clinical trials in patents with hematological malignancies or small cell lung cancer [47], [48]. In the present study we used the VXL treatment regimen in combination with a low dose of ABT-737 in order to assess its applicability as a platform for testing of novel drugs. The incorporation of ABT-737 into the VXL combination resulted in therapy that was well tolerated, thus providing evidence that additional drugs can be administered in conjunction with VXL treatment using this ALL xenograft model. Furthermore, in agreement with previously published data [23] we have shown that ABT-737 potentiated the effects of VXL therapy in resistant ALL xenografts, again strengthening the case for the incorporation of its clinical equivalent into multi-agent clinical trials in pediatric ALL patients. While delineating the mechanisms responsible for synergistic interactions between VXL and ABT-737 is beyond the scope of this study, we have previously suggested that downregulation of Mcl-1 following ASP treatment is a contributing factor [24].

A recent *in vitro* study utilizing patient samples has suggested that combining low dose ATO with DEX may improve the treatment of DEX-resistant ALL [25]. In the current study we combined VXL with ATO and tested its efficacy against several DEX resistant ALL xenografts *in vivo* and found that ATO only marginally delayed leukemia progression in two out of four xenografts tested. Therefore, our data support the reported limited clinical activity of ATO against ALL as a single agent [49], and provide limited rationale for its incorporation into combination treatment regimens in this disease.

In summary, we have described the optimization of a clinically relevant and robust combination chemotherapy regimen of VXL in the ALL NOD/SCID xenograft model. Using this induction/re-induction-type regimen we have further demonstrated its applicability for screening novel drugs for the identification of synergistic interactions that may warrant their advancement into clinical trials in high risk and relapsed ALL patients.

## Materials and Methods

### *In vitro* cell culture and cytotoxicity assays

All cell culture was carried out at 37°C with 5% O_2_ (equivalent to the physiological hypoxia found in bone marrow) and 5% CO_2_ [50]. A cell line from human T-cell leukemia established from a child at relapse (COG-LL-317) and a human pre-B leukemia cells established from a child at diagnosis prior to therapy (COG-LL-319) were obtained from the Children's Oncology Group (COG) Cell Line and Xenograft Repository (www.cogcell.org) [23]. They were maintained in Iscove's Modified Dulbecco's Medium (Cambrex, Walkersville, MD) supplemented with 3 mM L-glutamine, 5 µg/mL insulin and 20% heat-inactivated fetal bovine serum (FBS). The pre-B ALL cell line RS4-11 and the T-lymphoblast ALL cell line CEM were cultured in RPMI-1640 medium (Mediatech Inc., Herdon, VA) supplemented with 10% heat-inactivated FBS.

The cytotoxic effects of VCR, DEX and ASP were determined using the DIMSCAN assay system [50]. The drug concentration ranges for the assay were 0.5–10 ng/mL for VCR, 50–500 nM for DEX and 1–10 U/mL for ASP at fixed ratios. Cells were treated over 48 h (RS4-11) or 72 h (CEM, COG-LL-317 and COG-LL-319), following which fluorescein diacetate and eosin Y were added to final concentrations of 10 mg/mL and 0.1% (w/v), respectively. Fluorescence was measured using digital image microscopy, and the fractional survival of treated cells was determined compared to that of controls. Combination Indices (CIs) were calculated using Calcusyn (Biosoft, Cambridge, UK) as previously described [50]. With this method, a CI<0.3 indicates strong synergy, 0.3–0.7 synergy, 0.7–0.85 moderate synergy, 0.85–0.9 slight synergy, 0.9–1.1 additivity, 1.1–1.2 slight antagonism, 1.2–1.45 moderate antagonism, 1.45–3.3 antagonism, >3.3 strong antagonism.

### Ethics statement

All mice were maintained under barrier conditions and experiments were conducted using protocols and conditions approved by the Animal Care and Ethics Committee of the University of New South Wales (ACEC: 04/124b and 07/157b).

### *In vivo* xenograft model of childhood ALL

NOD/SCID (NOD.CB17-*Prkdc^scid^*/J) mice at 6–8 weeks of age were obtained from the Institute of Medical and Veterinary Science, Adelaide, SA, Australia. The establishment of a panel of continuous xenografts from childhood ALL biopsies in NOD/SCID mice and assessment of their *in vivo* responses to DEX and VCR have previously been described in detail elsewhere [17], [18]. Patient demographics, cytogenetic and clinical data, from whom the xenografts were established are represented in Table S1. Leukemia engraftment was monitored by flow cytometric quantification of the proportion of human CD45-positive (huCD45^+^) cells versus total murine CD45^+^ cells in the PB, bone marrow, spleen and/or liver, as described previously [17], [18].

For *in vivo* drug treatments, groups of 6–8 mice were inoculated with 2.5–5×10^6^ ALL mononuclear cells purified from spleens of previously engrafted mice. When the median %huCD45^+^ cells in the PB reached 1%, mice were randomized to receive drug or vehicle treatment. All drugs were administered by intraperitoneal injection (i.p.). Drug schedules were as follows: VCR (Sigma-Aldrich, Inc., St. Louis, MO, U.S.A.) once a week for 4 weeks; DEX and ATO (Sigma-Aldrich, Inc.), ASP (Leunase®, Aventis, Lane Cove, NSW, Australia), or ABT-737 (kindly provided by Abbott Laboratories, IL, U.S.A.) Monday to Friday for 4 weeks. For VXL/ABT-737 combination treatments VXL and ABT-737 were administered 6–8 hours apart.

Event-free survival (EFS) was calculated for each mouse as the number of days following randomization (6 days before treatment initiation) until the %huCD45^+^ cells in the PB reached 25%, or until mice experienced drug or leukemia-related morbidity (weight loss, lethargy, ruffled fur). EFS values for different treatments were compared by Kaplan-Meier survival curves [51]. For comparisons between different xenografts and between responses to various drug treatments, the median EFS of control mice was subtracted from the median EFS of drug-treated mice to generate a leukemia growth delay (LGD).

Synergistic, additive, or antagonistic drug interactions *in vivo* were estimated theoretically by the summation of individual LGD values [52]. This method assumes that the delay in tumor progression induced by a defined period of drug treatment is proportional to the log cell kill. The combined effect of more than one drug is the summation of individual delay times, assuming independence, and an additive effect is obtained when the LGD for a combination of drugs is equal to the sum of LGDs for each individual drug. An observed LGD for a combination of drugs that is greater than the sum of the single agent's LGDs indicates synergy, while an observed delay less than the sum indicates antagonism.

### Pharmacokinetic study

NOD/SCID mice engrafted with ALL-19 were treated i.p. with single agents VCR (0.15 mg/kg), DEX (5 mg/kg), ASP (1000 U/kg) or a combination of these three drugs at the same doses. Three mice contributed one blood sample via terminal cardiac puncture at each of the following time points after drug administration: 0, 0.5, 1, 2, 4, 6, 12, and 24 h, and the plasma was stored at −80°C until analysis. Drug concentrations were measured using previously published methods validated in our laboratory (see Methods S1).

All concentration-time data from the single agent group of mice and the drug combination group of mice were analyzed simultaneously. Nonlinear mixed-effects modeling with NONMEM VII software [53] was used to fit an appropriate compartmental model to the concentration-time data. The effect of the drug combination was assessed by adding the drug combination as a covariate on the CL parameter and assessing the change in the objective function value. A decrease ≥3.8 units indicates that the covariate model significantly increases the model fit to the data (*p*>0.05).

### Statistical methods

Median EFS values were obtained for control and treated cohorts, the difference between these medians (LGD) was calculated for each treatment group. Kaplan-Meier survival curves [51] were generated based on the event definition of 25% of huCD45^+^ cells out of the number of total circulating leukocytes in PB. The exact log-rank test (using Graphpad Prism 5.02) was used to compare EFS distributions between treatment and control groups (two tailed). *P*-values≤0.05 were considered evidence of significant differences. The non-parametric Mann-Whitney test was used to compare non-normally distributed datasets. Two sided contingency testing (chi square) was used for testing interdependency of classification above or below a selected threshold of 55 days of LGD for xenografts generated from patient samples of good or poor clinical outcome.

## Supporting Information

Methods S1 **Detailed description of analytical methodology used for the pharmacokinetic study of the drugs VCR, DEX and ASP in mice (with references).**
(DOC)

Figure S1 **Time-course and tissue distribution of engraftment of ALL-19 in NOD/SCID mice.** Mice were inoculated with 5×10^6^ ALL-19 cells i.v. At weekly intervals two mice were culled and the %huCD45^+^ cells relative to total (human+murine) CD45^+^ cells were monitored in peripheral blood (open circles), spleen (closed squares), bone marrow (closed circles) and liver (open triangles) by flow cytometry.
(TIF)

Figure S2 ***In vivo*** **responses of BCP-ALL xenografts to ASP. Male NOD/SCID mice were inoculated with ALL-3 (A); ALL-7 (B); and ALL-19 (C) cells, monitored for engraftment, and treated with saline (dashed lines) or 2500 U/kg of ASP (solid lines).** Each line represents a single mouse. EFS represented by Kaplan-Meier analysis (**D**) for control (gray lines) or ASP-treated (black lines) mice, for ALL-3 (solid lines), ALL-7 (dotted lines) and ALL-19 (dashed lines). Shaded boxes represent ASP or vehicle treatment periods.
(TIF)

Figure S3 ***In vivo*** **responses of ALL-19 to moderate dose VCR, DEX and ASP.** Female mice were inoculated with ALL-19 cells, monitored for engraftment and treated with diluent (dashed lines) or with drugs (solid line): VCR (0.25 mg/kg) (**A**); DEX (7.5 mg/kg) (**B**); ASP (2500 U/kg) (**C**); or the combination of the three drugs (VXL) at the same doses (**D**). The %huCD45^+^ cells in PB was measured at weekly intervals. Kaplan-Meier analysis of EFS (**E**) of controls (gray dashed line), VCR treated (gray solid line), DEX treated (black dashed line), ASP treated (black dotted line) and VXL treated (black solid line) groups. The two early events in the VXL-treated group were not leukemia-related. Shaded boxes represent treatment period.
(TIF)

Table S1 **Detailed demographic, cytogenetic and clinical characteristics of the patients from whom biopsy samples were obtained for establishment of the different xenografts used in this study.**
(DOC)

Table S2 ***In vivo*** **responses of xenografts ALL-3, ALL-7 and ALL-19 induced by treatment with either VCR, DEX, ASP or their combination at different doses.** Median EFS and corresponding LGD (both in days) are shown.
(DOC)
