# Supplementary material for: Pharmacokinetic Modeling of an Induction Regimen for In Vivo Combined Testing of Novel Drugs against Pediatric Acute Lymphoblastic Leukemia Xenografts
Source: PLoS One. 2012 Mar 29;7(3):e33894. doi: 10.1371/journal.pone.0033894 (PMC3315513; doi:10.1371/journal.pone.0033894)
Supplement: Methods S1 — Detailed description of analytical methodology used for the pharmacokinetic study of the drugs VCR, DEX and ASP in mice (with references). (DOC) [file pone.0033894.s001.doc]

**Methods S1**

*Pharmacokinetic Study and Bioanalytical Methodology*

A validated HPLC-ESI-MS/MS method was used to quantify VCR murine plasma concentrations [S1,S2,S3]. Vinorelbine (Sigma-Aldrich), used as internal standard, was added to 50 µL of sample prior to liquid-liquid extraction. To each sample 1.5 mL of *tert*-butyl methyl ether was added and the solution was vortexed and centrifuged to separate the organic and aqueous layers. The organic supernatant was evaporated under house nitrogen and the dried sample was reconstituted with methanol/15 mM ammonium acetate with 0.02% formic acid (60/40, v/v). Reproducible chromatography was achieved with a Luna C8(2), 3µm, 75 x 2.0 mm column (Phenomonex, Torrance, CA, USA) and a mobile phase of methanol/15 mM ammonium acetate with 0.02% formic acid (77/23, v/v). An API 3000 mass spectrometer (Applied Biosystems, Carlsbad, CA, USA) equipped with an electrospray source in the positive-ion multiple reaction monitoring (MRM) mode was used for detection. The MRM transition monitored for VCR was *m/z* 825.4 to *m/z* 765.4.

DEX in murine plasma was quantified with a validated HPLC analytical method using ultraviolet absorbance detection [S4]. An internal standard of triamcinolone acetonide (Sigma-Aldrich) was added to samples followed by a simple solid-phase extraction procedure with Strata-X (30 mg) columns (Phenomenex). The extraction columns were pre-treated with 1.0 mL of methanol, equilibrated with 1.0 mL of double distilled water, loaded with sample, washed with 2 x 1.0 mL of 5% methanol in water, and eluted with 100% methanol. Extracted samples were dried under house nitrogen and reconstituted with 20% methanol in water. Analytic separation was achieved with a Luna 3 µm, C18(2), 75 x 2.0 mm column (Phenomenex) and a 0.1% formic acid in water/acetonitrile/iso-propanol (76/19/5, v/v/v) mobile phase. DEX was detected with an SPD-20A (Shimadzu, Tokyo, JPN) at 254 nm.

The concentration of ASP in mouse plasma was indirectly quantified by spectrophotometric detection of the enzymatically initiated oxidation of reduced nicotinamide adenine dinucleotide (NADH). Briefly, 10 µL of mouse plasma were mixed with 190 µL solution of ASP, α-ketoglutaric acid, L-glutamic oxaloacetate transaminase, L-malic dehydrogenase, and β-NADH in 0.05 M Tris-HCl/glycerol (80/20, v/v) buffer, pH 8.45. All chemicals were provided by Sigma-Aldrich. The kinetic decrease of NADH was measured by absorbance of 340 nm over 10 min with a µQuant spectrophotometer (Bio-Tek, Winooski, VT, USA) [S5,S6,S7,S8,S9].

**Supplementary References**

S1. Guo P, Wang X, Zhou F, Gallo JM, Guo P, et al. (2004) Determination of vincristine in mouse plasma and brain tissues by liquid chromatography-electrospray mass spectrometry. J Chromatogr B: Anal Technol Biomed Life Sci 809: 273-278.

S2. Guilhaumou R, Solas C, Rome A, Giocanti M, Andre N, et al. (2009) Validation of an electrospray ionization LC/MS/MS method for quantitative analysis of vincristine in human plasma samples. J Chromatogr B: Anal Technol Biomed Life Sci 878: 423-427.

S3. Damen CW, Rosing H, Tibben MM, van Maanen MJ, Lagas JS, et al. (2008) A sensitive assay for the quantitative analysis of vinorelbine in mouse and human EDTA plasma by high-performance liquid chromatography coupled with electrospray tandem mass spectrometry. J Chromatogr B: Anal Technol Biomed Life Sci 868: 102-109.

S4. Yang L, Panetta JC, Cai X, Yang W, Pei D, et al. (2008) Asparaginase may influence dexamethasone pharmacokinetics in acute lymphoblastic leukemia. J Clin Oncol 26: 1932-1939.

S5. Asselin BL, Whitin JC, Coppola DJ, Rupp IP, Sallan SE, et al. (1993) Comparative pharmacokinetic studies of three asparaginase preparations. J Clin Oncol 11: 1780-1786.

S6. Berg SL, Balis FM, McCully CL, Godwin KS, Poplack DG (1993) Pharmacokinetics of PEG-L-asparaginase and plasma and cerebrospinal fluid L-asparagine concentrations in the rhesus monkey. Cancer Chemoth Pharm 32: 310-314.

S7. Jayaram HN, Cooney DA, Jayaram S, Rosenblum L (1974) A simple and rapid method for the estimation of L-asparaginase in chromatographic and electrophoretic effluents: comparison with other methods. Anal Biochem 59: 327-346.

S8. Tsurusawa M, Chin M, Iwai A, Nomura K, Maeba H, et al. (2004) L-Asparagine depletion levels and L-asparaginase activity in plasma of children with acute lymphoblastic leukemia under asparaginase treatment. Cancer Chemoth Pharm 53: 204-208.

S9. Wolf M, Wirth M, Pittner F, Gabor F (2003) Stabilisation and determination of the biological activity of L-asparaginase in poly(D,L-lactide-co-glycolide) nanospheres. Int J Pharm 256: 141-152.
