# Supplementary figures and images for: Pharmacokinetic Modeling of an Induction Regimen for In Vivo Combined Testing of Novel Drugs against Pediatric Acute Lymphoblastic Leukemia Xenografts
Source: PLoS One. 2012 Mar 29;7(3):e33894. doi: 10.1371/journal.pone.0033894 (PMC3315513; doi:10.1371/journal.pone.0033894)

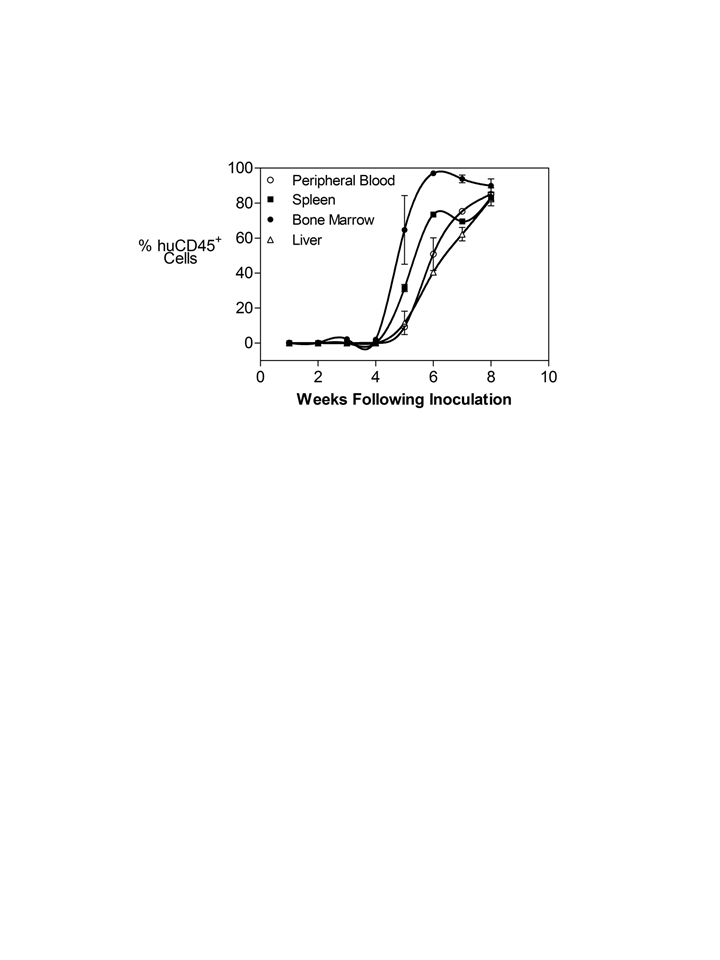

Supplement: Figure S1 — Time-course and tissue distribution of engraftment of ALL-19 in NOD/SCID mice. Mice were inoculated with 5×10^6^ ALL-19 cells i.v. At weekly intervals two mice were culled and the %huCD45+ cells relative to total (human+murine) CD45+ cells were monitored in peripheral blood (open circles), spleen (closed squares), bone marrow (closed circles) and liver (open triangles) by flow cytometry. (TIF) [file pone.0033894.s002.tif]

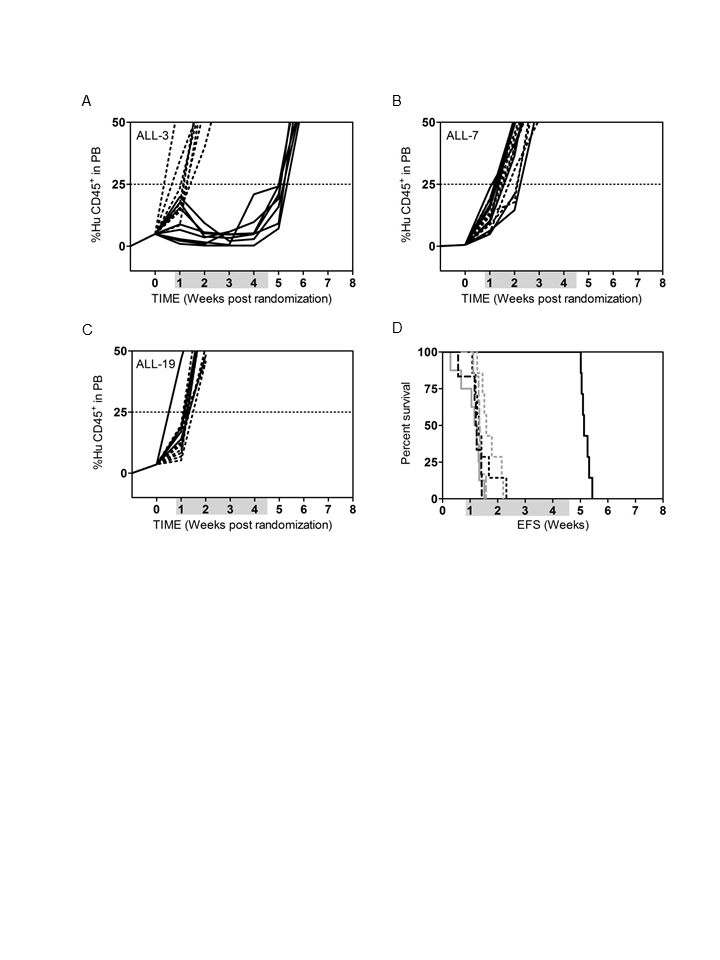

Supplement: Figure S2 — In vivo responses of BCP-ALL xenografts to ASP. Male NOD/SCID mice were inoculated with ALL-3 (A); ALL-7 (B); and ALL-19 (C) cells, monitored for engraftment, and treated with saline (dashed lines) or 2500 U/kg of ASP (solid lines). Each line represents a single mouse. EFS represented by Kaplan-Meier analysis (D) for control (gray lines) or ASP-treated (black lines) mice, for ALL-3 (solid lines), ALL-7 (dotted lines) and ALL-19 (dashed lines). Shaded boxes represent ASP or vehicle treatment periods. (TIF) [file pone.0033894.s003.tif]

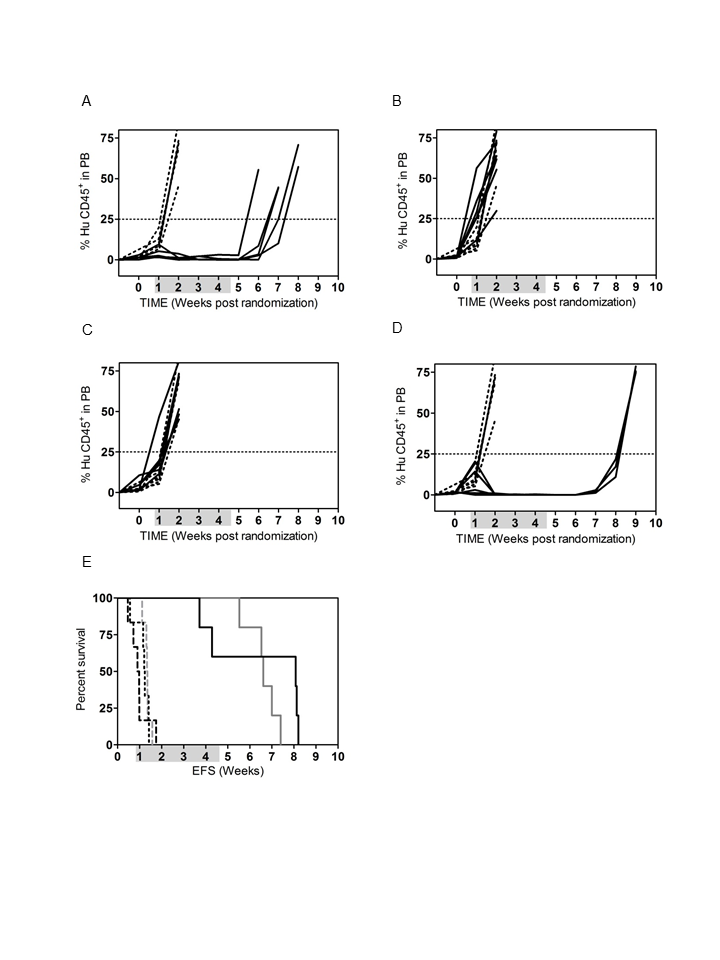

Supplement: Figure S3 — In vivo responses of ALL-19 to moderate dose VCR, DEX and ASP. Female mice were inoculated with ALL-19 cells, monitored for engraftment and treated with diluent (dashed lines) or with drugs (solid line): VCR (0.25 mg/kg) (A); DEX (7.5 mg/kg) (B); ASP (2500 U/kg) (C); or the combination of the three drugs (VXL) at the same doses (D). The %huCD45+ cells in PB was measured at weekly intervals. Kaplan-Meier analysis of EFS (E) of controls (gray dashed line), VCR treated (gray solid line), DEX treated (black dashed line), ASP treated (black dotted line) and VXL treated (black solid line) groups. The two early events in the VXL-treated group were not leukemia-related. Shaded boxes represent treatment period. (TIF) [file pone.0033894.s004.tif]
