# Supplementary material for: Pharmacokinetic Modeling of an Induction Regimen for In Vivo Combined Testing of Novel Drugs against Pediatric Acute Lymphoblastic Leukemia Xenografts
Source: PLoS One. 2012 Mar 29;7(3):e33894. doi: 10.1371/journal.pone.0033894 (PMC3315513; doi:10.1371/journal.pone.0033894)
Supplement: Table S1 — Detailed demographic, cytogenetic and clinical characteristics of the patients from whom biopsy samples were obtained for establishment of the different xenografts used in this study. (DOC) [file pone.0033894.s005.doc]

**Table S1**. Patients’ demographic, cytogenetic and clinical details.

| **Xenograft** | **Age at diagnosis** | **ALL subtype** | **Disease status** | **Cytogenetics of Original Patient Biopsy** | **Length of CR1** | **Site of relapse** | **Survival after relapse 1** | **Current clinical status** |
| --- | --- | --- | --- | --- | --- | --- | --- | --- |
|  | (months)/sex |  |  |  | (months) |  | (months) |  |
|  |  |  |  |  |  |  |  |  |
| **ALL-2** | 65/F | c-ALL | Relapse 3 | 46, XX, NAD | 30 | BM/CNS | 46 | DOD |
| **ALL-3** | 154/F | Pre-B | Diagnosis | 46, XX, del(11;q23) | 38 | BM | 172 | CR2 |
| **ALL-4** | 105/M | Ph^+^,c-ALL | Diagnosis | 9q34(abl x2), 22q11 (bcr x2)(abl con bcr x1)/9q34(abl x2),22q11(bcr x2) | 10 | BM | 1 | DOD |
| **ALL-7** | 88/M | Biphenotypic | Diagnosis | 46, XY, t(17;19)(q21;q13) | 7 | BM | 6 | DOD |
| **ALL-8** | 152/M | T-ALL | Relapse 1 | 46, XY, NAD | 17 | BM | 1 | DOD |
| **ALL-10** | 48/M | c-ALL | Diagnosis | 47, XY, +mar(12)/ 46, XY (13) | 98 | - | - | CR1 |
| **ALL-11** | 37/F | c-ALL | Diagnosis | 46, XX, del(12p13), del(13q12) (8)/ 46, XX (12) | 137 | - | - | CR1 |
| **ALL-16** | 122/F | T-ALL | Diagnosis | 46, XX, NAD | 120 | - | - | CR1 |
| **ALL-17** | 107/F | c-ALL | Diagnosis | Not done | 25 | CNS | 103 | CR2 |
| **ALL-19** | 194/M | c-ALL | Relapse 1 | 46, XY, NAD | 4 | BM | 7 | DOD |

**c-ALL**, common (CD10^+^) pre-B ALL; **NAD**, no abnormality detected; **Ph^+^**,c-ALL, Philadelphia chromosome-positive c-ALL; **Pre-B**, B-cell precursor ALL; **BM**, bone marrow; **CNS**, central nervous system; **CR**, complete remission; **DOD**, died of the disease.
