# Supplementary material for: Pharmacokinetic Modeling of an Induction Regimen for In Vivo Combined Testing of Novel Drugs against Pediatric Acute Lymphoblastic Leukemia Xenografts
Source: PLoS One. 2012 Mar 29;7(3):e33894. doi: 10.1371/journal.pone.0033894 (PMC3315513; doi:10.1371/journal.pone.0033894)
Supplement: Table S2 — In vivo responses of xenografts ALL-3, ALL-7 and ALL-19 induced by treatment with either VCR, DEX, ASP or their combination at different doses. Median EFS and corresponding LGD (both in days) are shown. (DOC) [file pone.0033894.s006.doc]

**Table S2. *In vivo* responses of ALL-3, ALL-7 and ALL-19 to VCR, DEX and ASP.**

| **TREATMENT** | | |  | **ALL-3** | | **ALL-7** | | **ALL-19** | |
| --- | --- | --- | --- | --- | --- | --- | --- | --- | --- |
| **VCR** | **DEX** | **ASP** |  | **Median EFS** | **LGD** | **Median EFS** | **LGD** | **Median EFS** | **LGD** |
| **(mg/kg)** | **(mg/kg)** | **(U/kg)** |  | **(days)** | **(days)** | **(days)** | **(days)** | **(days)** | **(days)** |
| 0.25 | - | - |  | - | - | 60.9 | 34.1 | 46.3 | 36.9 |
| 0.15 | - | - |  | - | - | - | - | 26.3 | 19.4 |
| - | 7.5 | - |  | - | - | 56.9 | 30.2 | 6.6 | 0 |
| - | 5 | - |  | 126.8 | 105.8 | - | - | 8.5 | 1.6 |
| - | - | 2 500 |  | 35.9 | 27.5 | 9.2 | 0 | 8.5 | 0 |
| - | - | 1 000 |  | - | - | - | - | 7.6 | 0.7 |
| 0.25 | 7.5 | 2 500 |  | - | - | 109.5 | 82.8 | 56.9 | 47.5 |

The median EFS of control mice were 8.7 days for ALL-3, 26.7 days for ALL-7 and 6.9 or 9.4 days for ALL-19, respectively.
